# Sleep disturbances in the Croatian adult population amidst the COVID-19 pandemic

**DOI:** 10.1192/j.eurpsy.2023.871

**Published:** 2023-07-19

**Authors:** I. Miskulin, I. Fotez, M. Miskulin, M. Kristic

**Affiliations:** 1 Department of Public Health, Faculty of Medicine Osijek; 2 Department of Clinical Medicine, Faculty of Dental Medicine and Health Osijek, Osijek; 3 Department of Psychiatry, University Hospital Split, Split, Croatia

## Abstract

**Introduction:**

It has been shown that various traumatic events, such as social isolation connected with the COVID-19 pandemic, can produce psychological distress and anxiety symptoms which negatively impact sleep quality.

**Objectives:**

This study aimed to investigate the influence of the COVID-19 preventive measures, especially social isolation, on sleep quality of the Croatian adult population during the COVID-19 pandemic.

**Methods:**

This cross-sectional questionnaire study was conducted from February to June 2021 period. A validated, anonymous questionnaire that contained questions regarding demographic data, as well as Pittsburgh Sleep Quality Index (PSQI) was self-administered to a convenient sample of Croatian adults from central and northwestern Croatia.

**Results:**

The study sample included 939 subjects with, median age of 42 years (interquartile range 35-48), 35.4% males, and 64.6% females. According to the PSQI there were 22.6% of subjects who presented sleep disturbances. Sleep disturbances were more frequent among females (p<0.001), inhabitants of the Croatian capital Zagreb (p=0.001), subjects who were not infected with COVID-19 virus (p=0.042), subjects who had fear of coronavirus infection in the workplace (p<0.001), subjects who had fear of coronavirus infection during daily life activities (p<0.001), subjects who had fear of coronavirus infection during daily physical activities (p<0.001) and subjects who worked with limited social contact (p=0.005).

**Conclusions:**

The COVID-19 pandemic has a significant negative influence on the sleep quality of the Croatian general population. Development of appropriate supportive programs that enhance mental health and sleep quality during pandemics is needed to address mental health problems in Croatia during the ongoing pandemic.

**Disclosure of Interest:**

None Declared

